# Risk factors of thrombosis in Chinese subjects with acute promyelocytic leukemia

**DOI:** 10.1186/s12959-021-00294-7

**Published:** 2021-06-15

**Authors:** Xueya Zhang, Xizhe Guo

**Affiliations:** Department of Hematology, the Second Affiliated Hospital of Fujian Medical University, 34 Zhongshan North Road, Quanzhou, 362000 Fujian Province China

**Keywords:** Acute promyelocytic leukemia, Thrombotic events, Khorana score, PAI-1, FLT3/ITD

## Abstract

**Background:**

Acute promyelocytic leukemia (APL) is a special type of acute myeloid leukemia Thrombosis is at increased risk complication in patients with this disease. However, the risk factors of thrombosis related to Chinese APL patients are not fully understood.

**Methods:**

In this study, clinical and laboratory data of 44 consecutively Chinese APL patients were collected and analyzed.

**Results:**

One arterial and 6 venous thrombosis occurred in 44 patients, including 22 males and 22 females, with a median age of 44 years (range from 18 to 74 years). The ratio of male and female gender, age, white blood cell count, hemoglobin, platelets, disease risk stratification, CD2, Khorana score, differentiation syndrome (DS) and gene mutation related to prognosis of APL, including DNMT3A, TET2, IDH1, IDH2, NRAS and ASXL1 in the two groups with and without thrombosis were not statistically significant. The detection rate of PAI-1 genotype 4G4G was 71.4% (5/7) in 7 patients with thrombosis, while the detection rate of PAI-1 genotype 4G4G in 37 patients without thrombosis was 8.1% (3/37). The differences between the two groups in WT-1 (*P* = 0.01), PAI-1 4G4G (*P* = 0.0009), bcr3 (*P* = 0.027), CD15 (*P* = 0.005), and FLT3-ITD mutation (*P* = 0.0008) were statistically significant. Using multivariate analysis, the risk factors of venous thrombosis in APL were CD15 (*P* = 0.043), PAI-1 4G4G (*P* = 0.009), WT-1 (*P* = 0.043) and FLT3/ITD (*P* = 0.013), respectively.

**Conclusion:**

Our results suggested the PAI-1 gene 4G4G type, CD15, WT-1 and FLT3-ITD mutations excluding DNMT3A, TET2, IDH1/2, NRAS and ASXL1 are risk factors of thrombotic events in Chinese APL patients.

## Introduction

Acute promyelocytic leukemia (APL) is a special type of acute myeloid leukemia with cytogenetic characteristics of t (15; 17) (q22; q21) formation the PML/RARa fusion gene [1–3]. APL is a kind of leukemia with dangerous prognosis in early phase [4]. Clinical manifestations of acute leukemia, including anemia, hemorrhage, infection, hepatosplenomegaly, lymphadenopathy, bone pain, etc. [5, 6], are commonly found in APL. Among them, bleeding tendency is the most significant clinical manifestation of APL patients, and also the primary factor for early death of APL patients [7–10]. In addition, the incidence of thrombotic events (TE) in APL is higher than that in other types of leukemia, and the incidence of arterial and venous thrombosis is reported to be between 2% and 10–15% according to previous data documented [11–14]. APL venous thrombosis can occur in deep veins, cerebral venous sinus, portal vein, and hepatic vein, while APL arterial thrombosis occurs mostly in peripheral arterial occlusion, myocardial infarction and ischemic stroke [15].

Therefore, it is very important to evaluate the risk of thrombosis in the early stage of acute promyelocytic leukemia, especially in the process of induced differentiation and other treatment, which helps clinical doctors provide intervention treatment to reduce complications.

At present, with regard to the risk factors of APL related thrombotic events, limited research results showed that they could be related to high leukocyte, CD2/CD15 positive, FLT3/ITD positive, PML/RARa fusion gene variant, retinoic acid syndrome, high platelet count and male [16–18]. However, the epidemiology and risk factors of APL related thrombotic events have not been fully discovered. Thus, through a single center retrospective study, we characterized the clinical information of 44 patients with newly diagnosed APL, analyzed the risk factors of APL related thrombotic events, and provided strategies for better clinical treatment of APL patients.

## Patients and methods

Forty-four newly diagnosed APL patients were consecutively enrolled from Department of Hematology of the Second Affiliated Hospital of Fujian Medical University from January 2013 to December 2019. The study was approved by informed consent of patients and hospital ethics committee. All patients were diagnosed by cytomorphology, immunophenotyping, cytogenetics, and molecular biology, including conventional G-banding assay for t (15;17) (q22; q12), reverse transcription polymerase chain reaction (RT-PCR) for detection of PML-RARA fusion transcripts. The FLT3/ITD and WT1 gene mutations and epigenetic modifier genes (EMG) mutations related to prognosis of APL were detected in 44 patients by PCR and next generation sequencing (NGS). Four color flow cytometry (FACS-calibur, BD, USA) was used to detect the surface antigens of leukemia cells. Monoclonal antibodies were HLA-DR, CD2, CD3, CD4, CD5, CD7, CD8, CD9, CD10, CD11b, CD13, CD14, CD15, CD16, CD19, CD20, CD22, CD33, CD34, CD38, CD56, CD64, CD71, CD117, CD123, MPO and CD45.

All patients were treated with all-trans retinoic acid plus anthracyclines and/or arsenic trioxide. During the induction treatment, the platelet count of patients was controlled above 30 × 10^9^/L by infusion of platelets, and the fibrinogen level was controlled above 1.5 g/L by fresh frozen plasma infusion. The patients did not use antifibrinolytic drugs, such as tranexamic acid, and did not have a central venous catheter during the induction therapy.

### Detection of coagulation related parameters

The activated prothrombin time (APTT), prothrombin time (PT) and fibrinogen were detected by Automatic Coagulation Analyzer (Sysmex Corporation, Japan). D-dimer was measured by immunoturbidimetry. The normal range of them is shown as follows: APTT 25–35 s, PT 6–14 s, fibrinogen 2–4 g /L and D-dimer 0–0.5 μg/ml.

### Detection of plasminogen activator inhibitor-1 (PAI-1) gene polymorphism

Plasminogen activator inhibitor-1 (PAI-1) gene 4G/5G polymorphism was performed in 7 APL patients with TE and 37 APL patients without TE as previously described [19].

### Detection of thrombosis and bleeding events

From the day of admission to our department to the last day of follow-up, the thrombotic events and bleeding of APL patients were evaluated and recorded. Bleeding severity classification criteria was assessed using the International Society on Thrombosis and Haemostasis (ISTH) bleeding assessment tool [20]. Symptomatic or asymptomatic thrombosis originating from vein and artery was confirmed by imaging techniques. Such as, deep venous thrombosis (DVT) and cerebral infarction was performed by Color Doppler Vascular Ultrasound and Computed Tomography (CT) or Magnetic Resonance Imaging (MRI) image, respectively.

### Treatment of thrombotic diseases

In the acute phase of venous thrombosis, under the condition of ensuring platelets value > 30 × 10^9^/L and fibrinogen > 1.5 g/l, patients received the intravenous injection of heparin for 2 weeks and then received warfarin sequential therapy for another 3 months. During the short-term treatment of thrombosis, the warfarin dosage was adjusted based on the international normalized ratio (INR) value. Antiplatelet drugs were used in the treatment of arterial thrombosis, and the platelet count was maintained > 100 × 10^9^/L during treatment. Three months of anticoagulation and antiplatelet therapy, the recanalization of blood vessels of patients would be detected by Color Doppler Ultrasound and CT or MRI image [20, 21].

### Statistical analysis

We used chi square and Fisher’s exact method to analyze the relationship between variables, by which *P* < 0.05 is considered statistically significant. The differences between groups were analyzed by log rank test. Univariate Cox proportional regression model and Multivariate Cox Proportional Regression model were used to identify risk factors. The odds ratio (OR) and 95% confidence interval (CI) were calculated by social science statistical software package.

## Results

The study population included 44 APL patients, 22 (50%) males and 22 (50%) females with a median age of 42 years (range 18–74 years). These population were all newly diagnosed APL patients admitted to our department from January 2013 to December 2019 and received treatment according to the PETHEMA APL99 protocol. Among them, 7 APL patients had thrombotic events, none of them had previous history of thrombosis and family history of thrombosis. One patient had arterial thrombosis (2.3%) and 6 patients had venous thrombosis (13.6%). No APL patients showed the symptoms of bleeding and thrombosis simultaneously. The clinical characteristics of APL patients with thrombotic events were shown in Table 1.

**Table 1:** Presenting characteristics of APL patients who developed thrombotic events

Characteristics of Patients	Patient 1	Patient 2	Patient 3	Patient 4	Patient 5	Patient 6	Patient 7
Age(years)/sex	27/M	27/F	22/M	45/M	53/F	55/M	67/F
WBC(×10^9^/L)	3.7	2.5	3.7	106.8	14.5	0.6	3.9
HGB(g/L)	56	59	99	46	57	67	71
PLT(×10^9^/L)	9	36	2	7	24	62	19
Risk stratification	Intermediate	Intermediate	Intermediate	High	High	Low	Intermediate
PML/RARα isoforms	Bcr1	Bcr3	Bcr1	Bcr3	Bcr3	Bcr3	Bcr3
Leukemic cell biomarks	CD2-	CD2-	CD2-	CD2-	CD2-	CD2-	CD2-
	CD15-	CD15+	CD15+	CD15+	CD15+	CD15+	CD15+
WT1 mutation status	negative	positive	negative	negative	positive	positive	positive
FLT3/ITD mutation status	negative	positive	positive	positive	positive	positive	positive
PAI-1 gene polymorphism	4G4G	4G5G	4G5G	4G4G	4G4G	4G4G	4G4G

### Thrombotic events in patients with APL

The rate of arterial thrombosis was 1/44 (2.3%), this patient was a female, aged 53 years, when the disease was first diagnosed, she felt weakness of the limbs. There was no history of thrombosis, hypertension, diabetes and hyperlipidemia of the female patient. The cerebral infarction of the patient was confirmed by CT and MRI, and then she was treated with antiplatelet drug. The rate of VTE was 6/44 (13.6%), the ratio of male to female was 4/2, and the median age was 37 years (22–67 years). Venous thrombosis occurred in 6 cases of lower extremity venous thrombosis, among which 4 cases occurred on the left and 2 cases on the right. All of those cases occurred during the induction of all-trans retinoic acid after diagnosed. The median time to diagnosis of thrombotic events was 14 days (9–26 days). Six patients with venous thrombosis were treated with unfractionated heparin (UFH) anticoagulation under the premise of platelet and fibrinogen supplementation and treated with sequential administration of warfarin later. After anticoagulant therapy for 2 weeks, the symptoms and signs related to thrombosis were improved. Moreover, serious bleeding and recurrent TE have not been observed during the 3 months of anticoagulant therapy.

The clinical characteristics of the two groups of APL patients with thrombotic events were shown in Table 2. There was no significant difference between the two groups in gender ratio (*P* = 0.68), age (*P* = 0.823), white blood cell count (*P* = 0.077), hemoglobin (*P* = 0.409), platelet (*P* = 0.334), disease risk stratification (*P* = 0.475), CD2 (*P* = 0.737) and differentiation syndrome (*P* = 0.562). There were significant differences between the two groups in PAI-1 4G4G (*P* = 0.0009), bcr3 (*P* = 0.027), CD15 (*P* = 0.005). To demonstrate that whether additional gene mutations involved in thrombotic events in APL patients, we analyzed the results of gene mutation related to prognosis of APL (See Table 3). There were significant differences between the two groups in WT-1 (*P* = 0 .01) and FLT3-ITD muations (*P* = 0.0008), excluding DNMT3A (*P* = 0.44), TET2 (*P* = 0.43), IDH1 (*P* = 0.6), IDH2 (*P* = 0.66), NRAS (*P* = 0.66), ASXL1 (*P* = 0.9). In Multivariate Cox Proportional Regression, as shown in Table 4, the risk factors of venous TE were CD15 (*P* = 0.043, OR 17.35, 95% CI 1.093–275.406), WT-1 (*P* = 0.043, OR 9.14, 95% CI 1.072–77.825) and FLT3-ITD mutations (*P* = 0.013, OR 21.89, 95% CI 1.938–247.176), respectively. As shown in Table 5, a total of 2 (4.5%), 16 (36.4%), 24 (54.5%), 2 (4.5%) of APL patients had a Khorana score of 0, 1, 2, 3, respectively. However, there were no statistically significance between two groups with thrombosis and without thrombosis.

**Table 2:** Comparison of characteristics in APL patients with and without thrombosis

Characteristics	APL patients with thrombosis(7)	APL patients without thrombosis(37)	*P* values
**Gender**			
Female	3	19	0.68
Male	4	18	0.68
**Age**(years)	42.3	40.9	0.823
WBC(×10^9^/L)	26.240	10.846	0.077
HGB(g/L)	63.40	81.21	0.409
PLT(×10^9^/L)	15.60	33.71	0.334
**Risk stratification**			
Low-risk	1 (14.3%)	10 (27%)	0.475
Intermediate-risk	4 (57.1%)	24 (64.9%)	0.697
High-risk	2 (28.6%)	3 (8.1%)	0.245
**PML-RARα gene type**			
Bcr1	2 (28.6%)	26 (70.3%)	
Bcr2	0 (0%)	0 (0%)	
Bcr3	5 (71.4%)	10 (27.0%)	0.027
Rare	0 (0%)	1 (2.7%)	
**Leukemic cell biomarks**			
CD2 positive	2 (28.6%)	13 (35.1%)	0.737
CD15 positive	6 (85.7%)	11 (29.7%)	0.005
**PAI-1 gene polymorphism**			
4G4G	5 (71.4%)	3 (8.1%)	0.0009
4G5G	2 (28.6%)	25 (67.6%)	
5G5G	0 (0%)	9 (24.3%)	
Differentiation syndrome	2 (28.6%)	7 (18.9%)	0.562

**Table 3:** Comparison of gene mutation related to prognosis of APL patients with and without thrombosis

Gene mutations	APL patients with thrombosis(7)	APL patients without thrombosis(37)	*P* values
WT-1	5 (71.4%)	9 (24.3%)	0.01
FLT3-ITD	6 (85.7%)	8 (21.6%)	0.0008
DNMT3A	0 (0%)	3 (8.1%)	0.44
TET2	2 (28.6%)	6 (16.2%)	0.43
IDH1	1 (14.3%)	3 (8.1%)	0.6
IDH2	0 (0%)	1 (2.7%)	0.66
NRAS	0 (0%)	1 (2.7%)	0.66
ASXL1	1 (14.3%)	6 (16.2%)	0.9
TP53	0 (0%)	0 (0%)	–
RUNX1	0 (0%)	0 (0%)	–
NPM1	0 (0%)	0 (0%)	–
CEBPA	0 (0%)	0 (0%)	–
C-kit	0 (0%)	0 (0%)	–
dupMLL	0 (0%)	0 (0%)	–

**Table 4:** Multivariate analysis using CD15 positive and PAI-1 gene 4G4G polymorphism in APL patients with and without thrombosis

Parameter	APL patients with thrombosis(7)	APL patients without thrombosis(37)	OR	CI (95%)	*P* values
CD15	6 (85.7%)	11 (29.7%)	17.35	1.093–275.406	0.043
PAI-1 4G4G	5 (71.4%)	3 (8.1%)	25.2	2.218–286.483	0.009
WT-1	5 (71.4%)	9 (24.3%)	9.14	1.072–77.825	0.043
FLT3/ITD	6 (85.7%)	8 (21.6%)	21.89	1.938–247.176	0.013

**Table 5:** Comparison of Khorana score in APL patients with and without thrombosis

Khorana score	APL patients with thrombosis(7)	APL patients without thrombosis(37)	*P* values
0	0 (0%)	2 (5.4%)	0.52
1	3 (42.9%)	13 (35.1%)	0.7
2	4 (57.1%)	20 (54.1%)	0.88
3	0 (0%)	2 (5.4%)	0.52

Additionally, risk factors of hereditary thrombophilia, including antithrombin III, protein C, protein S, antiphospholipid antibodies, lupus anticoagulant and homocysteine, were evaluated in these 7 APL patients with thrombotic events. However, these risk factors of thrombophilia were not identified. The detection rate of PAI-1 genotype 4G4G was 71.4% (5/7) in 7 APL patients with thrombotic events, while the detection rate of PAI-1 genotype 4G4G in APL patients without thrombotic events was 8.1% (3/37). There was significantly statistical difference between the two groups regarding whether thrombotic events occurred or not (*P* = 0.0009). Moreover, in Multivariate Cox Proportional Regression, the risk factor of venous thrombosis was PAI-1 4G4G (*P* = 0.009, OR 25.2, 95% CI 2.218–286.483), suggesting a high-risk factor related to thrombotic event.

## Discussion

Our results showed that the rate of APL arterial thrombosis was lower than that of venous thrombosis (2.3% vs 13.6%). Venous thrombosis occurred in the disease induction treatment phase. The median time of thrombotic events occurred 14 days (range 9–26 days). Six patients with venous thrombosis were given heparin anticoagulant therapy under the premise of platelet and fibrinogen supplementation, leading to the complete recanalization of the vein, while no serious bleeding events being observed. The rate of venous thrombosis is consistent with the data reported in the previous literature [16]. Other studies showed that the rate of APL related thrombotic events increased from 2% before the adoption of all-trans retinoic acid (ATRA) to 4.5–15% after the adoption of ATRA [22–24].

Currently, the risk factor of thrombosis in APL patients has not been fully discovered. Previous studies reported that these factors increased the risk of APL thrombosis, including male, high score performance status (PS), high white blood cell count and platelet count, low fibrinogen levels, hypoalbuminemia, PML/RARa fusion gene variant, CD2/CD15 and FLT3-ITD positive.

Our results showed that there was no significant difference between thrombotic events and gender ratio, age, white blood cell count, hemoglobin, platelets, disease risk stratification and CD2. In addition, PML/RARa (bcr3) and CD15 were statistically significant. Moreover, in Multivariate Cox Proportional Regression, the risk factor of venous thrombosis in APL was CD15 (*P* = 0.043, OR 17.35, 95% CI 1.093-275.406), suggesting high-risk factors associated with thrombotic events.

In order to further explore whether the risk factors for hereditary thrombosis are involved in the event of APL thrombosis, we detected antithrombin III, protein C, protein S, antiphospholipid antibodies and homocysteine in 7 APL patients with thrombotic events. However, the results showed no positive clinical significance, suggesting that thrombotic events in 7 APL patients did not involve genetic thrombophilia.

PAI-1 is a key regulator of endogenous fibrinolytic activity [25]. It is reported that the 4G/5G polymorphism of the PAI-1 gene affects plasma levels of PAI-1 [26]. The 4G/4G genotype is associated with higher plasma PAI-1 activity, which can lead to impaired fibrinolysis, thus increasing the risk of thrombosis [27, 28]. Previous study reported that PAI-1 gene 4G/4G in APL patients receiving ATRA treatment showed high PAI-1 level in vivo and increased the frequency of APL related thrombotic events [8]. Mitrovic M et al. demonstrated [16] that the PAI 4G/4G was five and two times more frequent in Serbia population with APL-related venous and arterial TE than in those without TE (*P* = 0.05). Similarly, results of this study showed that PAI-1 4G/4G was detected in 71.4% (5/7) of 7 patients with thrombosis, and PAI-1 gene polymorphism was 4G/5G (28.6%, 2/7) in 2 patients. The detection rate of PAI-1 4G/4G in the patients without thrombotic events was 8.1% (3/37), and the difference was statistically significant (*P* = 0.0009). In Multivariate Cox Proportional Regression, the risk factor of venous thrombosis was PAI-1 4G4G (*P* = 0.009, OR 25.2, 95% CI 2.218-286.483), suggesting a high-risk factor related to thrombotic event.

Thrombotic events are significant complications in malignant patients. Thrombosis risk is well defined in patients with solid tumors, and Khorana score is well validated for these patients. However, the value of Khorana scoring system in predicting the thrombotic events risk of hematological malignant diseases remains to be evaluated [29, 30]. Hence, we conducted a retrospective study to validate the use of the Khorana score for thrombotic events in APL patients. The results of our study showed that the Khorana score of all APL patients with thrombotic events were 1 and 2, and there was no significant difference between the two groups.

Lee YG et al. [31] suggested that advanced age and increasing cytogenetic risk were the independent risk factors of VTE after retrospectively analyzing 811 consecutive AML patients. The expression of additional gene mutations was correlated with the pathogenesis and prognosis of acute myeloid leukemia. Recently, APL patients with epigenetic modifier genes (EMG) mutations may exert negative impact on the overall survival and disease-free survival [32]. However, the possible of EMG mutations on the risk of VTE in APL patients has not been confirmed. We have analyzed the results of EMG mutations related to prognosis of APL. As shown in Table 3, there were significant differences between the two groups in WT-1 and FLT3-ITD mutations, excluding DNMT3A, TET2, IDH1, IDH2, NRAS and ASXL1. Moreover, in Multivariate Cox Proportional Regression, the risk factors of venous thrombosis in APL were WT-1 (*P* = 0.043, OR 9.14, 95% CI 1.072-77.825) and FLT3/ITD (*P* = 0.013, OR 21.89, 95% CI 1.938-247.176), respectively (Table 4). These results suggested that WT-1 and FLT3/ITD mutations were the risk factors of thrombotic events in APL patients.

## Conclusions

In conclusion, this study showed that the rate of thrombotic events in Chinese APL patients is consistent with the data reported previously. The WT-1 and FLT3/ITD mutations, PAI-1 gene 4G4G, CD15 positive expression are the risk factors of thrombotic events in Chinese APL patients. A better knowledge of risk factors of thrombosis is crucial to improve the management and outcome of thrombotic events in APL. However, the relatively small sample size and retrospective design are the limitations of this study. In addition, due to retrospective features, we have not performed a cumulative frequency analysis of thrombotic events in Chinese APL patients in our center, and it remains to be further explored in well designed clinical trials.
